# Untangling Photofaradaic and Photocapacitive Effects in Organic Optoelectronic Stimulation Devices

**DOI:** 10.3389/fbioe.2020.00284

**Published:** 2020-04-17

**Authors:** Vedran Ðerek, David Rand, Ludovico Migliaccio, Yael Hanein, Eric Daniel Głowacki

**Affiliations:** ^1^Laboratory of Organic Electronics, Campus Norrköping, Linköping University, Norrköping, Sweden; ^2^Wallenberg Centre for Molecular Medicine, Linköping University, Linköping, Sweden; ^3^Department of Physics, Faculty of Science, University of Zagreb, Zagreb, Croatia; ^4^Center of Excellence for Advanced Materials and Sensing Devices, Ruđer Bošković Institute, Zagreb, Croatia; ^5^Tel Aviv University Center for Nanoscience and Nanotechnology, School of Electrical Engineering Tel Aviv University, Tel Aviv, Israel; ^6^Faculty of Chemistry, Warsaw University of Technology, Warsaw, Poland

**Keywords:** bioelectronics, neurostimulation, organic electronics, photoelectrochemistry, photostimulation

## Abstract

Light, as a versatile and non-invasive means to elicit a physiological response, offers solutions to problems in basic research as well as in biomedical technologies. The complexity and limitations of optogenetic methods motivate research and development of optoelectronic alternatives. A recently growing subset of approaches relies on organic semiconductors as the active light absorber. Organic semiconductors stand out due to their high optical absorbance coefficients, mechanical flexibility, ability to operate in a wet environment, and potential biocompatibility. They could enable ultrathin and minimally invasive form factors not accessible with traditional inorganic materials. Organic semiconductors, upon photoexcitation in an aqueous medium, can transduce light into (1) photothermal heating, (2) photochemical/photocatalytic redox reactions, (3) photocapacitive charging of electrolytic double layers, and (4) photofaradaic reactions. In realistic conditions, different effects may coexist, and understanding their role in observed physiological phenomena is an area of critical interest. This article serves to evaluate the emerging picture of photofaradaic vs. photocapacitive effects in the context of our group’s research efforts and that of others over the past few years. We present simple experiments which can be used to benchmark organic optoelectronic stimulation devices.

## Introduction

**FIGURE 1:**
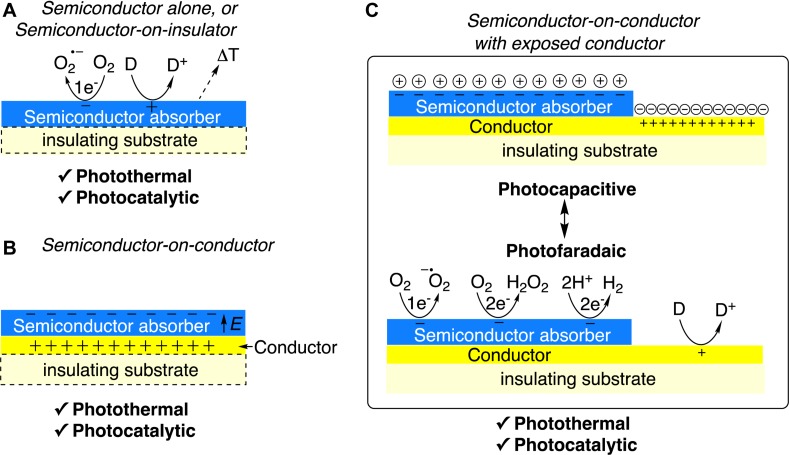
Light-induced effects at organic semiconductor/electrolyte interfaces. **(A)** Semiconductor layer as a standalone structure, or on an insulating support. Primarily photothermal or photochemical (catalytic) processes can occur. Reduction of oxygen is a likely process. **(B)** Semiconductor on a conductor. Photothermal and catalytic processes can occur here as well however, if the metal is sufficiently insulated from the electrolyte, no potential difference across the electrolyte will be induced; the electric field is localized within the solid-state layers. **(C)** Semiconductor on a conductor with the conductor also in direct contact with the electrolyte. With this arrangement, the back contact and the semiconductor establish closed-circuit conditions through the electrolyte: depending on the materials of choice either capacitive or faradaic interfaces can form (on one or both of the exposed parts of the device).

Light illumination of a semiconducting material (or heterostructure thereof) immersed in an electrolyte solution, with an energy greater than its band gap energy, can induce several different processes. What happens after light absorption depends on semiconductor material properties and heterostructure details. In Figure 1, we summarize a breakdown of three distinct device architectures, and the possible associated mechanisms of photophysiological coupling each can offer. In the following paragraphs, we describe these key photoeffects basing on literature examples.

### Photothermal Heating Effects

Organic semiconducting materials can be highly efficient light absorbers (Lin et al., 2012). Under the right conditions, this absorbed light can be converted into heat. In photovoltaic and photodiode technologies this conversion is highly undesired and band-gap engineering is used to avoid such processes. However, photothermal heating can also produce interesting and useful effects. For illumination timescales on the order of hundreds of milliseconds to seconds, light absorption causes a rise in local temperature that may have a physiological response. Heat-sensitive ion channels, such as the TRPV-family of ion channels, can be reversibly stimulated in this way (Albert et al., 2012). Human embryonic kidney (HEK) cells transfected with TRPV1 ion channels could be locally photothermally stimulated by irradiating (tens to hundreds of milliseconds) thin films of the polymeric semiconductor poly(3-hexylthiophene), P3HT (Lodola et al., 2017). In another example, organic nano-crystalline structures made from the material quinacridone also proved to be efficient, local, photothermal heaters (Sytnyk et al., 2017). These nano-structures form close and high surface-area interfaces with cultured cells. Local photothermal heating results in higher ion currents through open channels (potassium inward rectifier) which was demonstrated in irradiated rat basophilic leukemia cells growing on quinacridone nano-structures (5 mW/mm^2^, 100–800 ms) (Sytnyk et al., 2017). Photoactivation of cation influx through TRPV1 channels in HEK cells held at resting membrane potential was also shown for quinacridone/cell interfaces (30 μJ pulses) (Sytnyk et al., 2017). With high intensities of light at short timescales below a few ms, rapid heating can trigger a completely different mechanism: the local rise in temperature transiently increases cell membrane capacitance, generating a depolarizing current. The thermocapacitive stimulation effect was discovered and studied in detail by Shapiro, Bezanilla, and coworkers (Shapiro et al., 2012; Carvalho-de-Souza et al., 2015; Jiang et al., 2016). The magnitude of cell depolarization depends on the rate of temperature change ΔT/t (not on the absolute rise in temperature). Therefore, even though very high light intensities were used, they produced only harmless transient temperature changes. Shapiro et al. (2012) first detailed thermocapacitance for the case of NIR light around 1.5 μm wavelength, where water absorbs light and is heated directly. The thermocapacitive mechanism can be far more local and controlled when nano or microscale semiconducting particles located near a given cell can lead to selective stimulation of cells (Carvalho-de-Souza et al., 2015; Jiang et al., 2019). The effect can be achieved using a wide range of semiconductors (Zimmerman and Tian, 2018). A disadvantage of the method, however, is the necessity for powerful light sources. For chronic implants, the approach may be considered safe when short light pulses lead to low total dissipated energy. However, harnessing such high-power light intensities may prove challenging in many clinical applications.

### Photochemical Reactions

The next possible phenomenon of illuminated organic semiconductors in a physiological environment is the elicitation of a photochemical reaction. Photochemical reactions are precipitated by photogenerated electrons and holes. The former lead to the reduction of suitable electron-acceptors in solution, while the latter oxidize electron-donors. Possible reducible acceptors in physiological solutions are protons, dissolved dioxygen, or certain organic moieties such as quinone-containing molecules (Chowdhury et al., 2016). In the case of organic semiconductors (without suitable cocatalysts like Platinum), H_2_ evolution has proven to be inefficient (Bellani et al., 2018), and no evidence for H_2_ in a physiological context have been found. Oxygen reduction reactions, on the other hand, have been discovered to be highly favored on organic semiconductors (Bellani et al., 2015). The single-electron reduction of O_2_ to superoxide (Suppes et al., 2013; Gryszel et al., 2019) or the two-electron reduction to produce hydrogen peroxide (Jakešová et al., 2016; Węcławski et al., 2017; Gryszel et al., 2018b), H_2_O_2_, were demonstrated to proceed efficiently for several organic semiconductors. Both oxygen reduction reactions are thermodynamically more favorable than hydrogen evolution, with the two-electron peroxide reaction being 700 mV lower than H_2_ production. The dominance of the oxygen reduction reaction with organic semiconductors was shown in electrochemical (Warczak et al., 2018; Mitraka et al., 2019), photoelectrochemical (Jakešová et al., 2016; Gryszel et al., 2018a), and photochemical experiments (Gryszel et al., 2018b, 2019) for a wide range of organic semiconductors, including polythiophenes like P3HT, the biopolymer melanin (Migliaccio et al., 2018), and various carbonyl pigments (Gryszel et al., 2018a). The oxygen reduction products are considered reactive oxygen species (ROS) and have numerous physiological effects ranging from toxicity at high concentrations (Huang et al., 2013) to ion channel modulation (Gamper et al., 2006) and signaling effects (Lim et al., 2016) at low concentrations. In photochemistry involving photogenerated carriers, both electrons and holes must be consumed in order to sustain the process. In organic semiconductors, the fate of the photogenerated holes is often the critical element. Gryszel et al. have shown that various molecules can serve as sacrificial electron donors, such as oxalate, ethanol, and glucose (Gryszel et al., 2018b, 2019). In some cases where the highest occupied molecular orbital (HOMO) is sufficiently deep, oxidation of water as the donor is possible. Unfortunately, often self-oxidation of the organic semiconductor itself will serve to complete the photochemical cycle and lead to irreversible corrosion of the organic semiconductor (Gryszel et al., 2018b; Migliaccio et al., 2018). It was experimentally found that the deeper the HOMO, the more stable the semiconductor is with respect to self-oxidation. Quantifying the semiconductor oxidation degree, to benchmark the stability/degradation, is an important consideration. Gryszel et al. report a reliable method based on the redissolution of the semiconductor into a suitable solvent to obtain solutions that follow the Beer-Lambert law in UV-Vis absorbance (Gryszel et al., 2018b). It is then possible to calculate, based on the absorbance of such solutions, the quantity of degraded material. Registering absorption of the semiconductor in the solid state can give misleading results, as the optical density of a solid-state sample may remain high. An interesting example case is P3HT. Upon illumination in physiological solutions, P3HT thin films or colloidal particles have been demonstrated to photochemically reduce oxygen to produce ROS, ultimately yielding H_2_O_2_ as a metastable and easily quantifiable product. The ROS generation by P3HT has been exploited by Antognazza et al. to yield light-induced physiological effects in both *in vitro* (Moros et al., 2018; Antognazza et al., 2019; Lodola et al., 2019) and *in vivo* models (Tortiglione et al., 2017). When evaluated by UV-V is spectroscopy, P3HT throughout these experiments affords a negligible drop in solid-state absorbance, suggesting that the P3HT is stable under these conditions. However, using the Beer-Lambert method, whereby photochemically-aged films are redissolved and measured in chlorobenzene, it is possible to detect quantifiable degradation. Indeed, catalytical turn-over number (TON) of mol H_2_O_2_ produced divided by mol P3HT degraded is ≤ 1. Meaning that for every equivalent of ROS produced, a monomer of P3HT is consumed (Gryszel et al., 2018b). Various organic semiconductors have been tested for their TON under similar conditions, and compounds with deeper HOMO levels achieve TON of 10^2^–10^3^ range. However, this indicates that the irreversible oxidation of organic semiconductors is an issue that requires careful consideration. The fate of the oxidation products should be evaluated as this may also have important physiological consequences.

### Photocapacitive and Photofaradaic Currents

**FIGURE 2:**
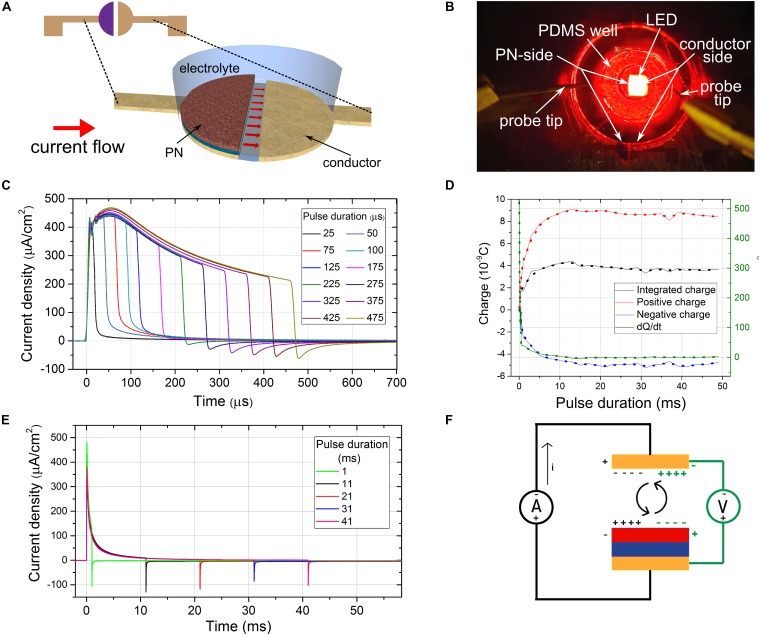
Measuring time-resolved photocurrents of a floating optoelectronic stimulation device. **(A)** The split-architecture OEPC structure. Red arrows signify the current flow lines. **(B)** The measurement configuration showing the elastomer electrolyte-containment well on top of a split-OEPC device, with red LED light source in the background **(C)** current traces of a split-OEPC device under light excitation with sub-1 ms light pulses, 33 mW/cm^2^ intensity. **(D)** Total charge, charge delivered during the positive and negative current phase, and time derivative of the total charge delivered for long light pulses **(E)** current traces of a split-OEPC device under light excitation with pulses longer than 1 ms. **(F)** A proposed model of a floating OEPC charge dynamics in darkness (green) and under light excitation (black).

Photoinduced charges can charge the surface of the semiconductor resulting in an electrolytic double-layer effect. Alternatively, this charge can be transferred to the solution to generate a product in a faradaic reaction. The photocapacitive effect is important for effective and safe coupling to cells, avoiding some of the chemical processes mentioned above. To generate substantial charging of the surface, an energetic asymmetry in the semiconductor structure is needed. If a semiconductor heterostructure has a built-in spatial asymmetry (resulting from doping or a Schottky contact) then photoexcitation can result in carrier generation followed by their spatial separation, leading to a gradient of potential across the surface of the semiconductor (Willner and Katz, 2005). The spatial separation of charges within the device is critical to generate a potential difference that will affect the surrounding medium. To achieve this, careful engineering of a particle or a film must be carried out; otherwise, no electric potentials in the solution can develop. Utilizing conductor/organic semiconductor structures drastically affects charge separation and localization. Conductor/organic interface devices can be divided into two categories: (1) buried conductor, where the conductor does not make contact with the electrolyte (Figure 1B) and (2) extended and exposed metal (Figure 1C). In the former case, the presence of the conductor can drive charge separation and localization. On the other hand, if the conductor is passivated from the electrolyte by the semiconductor layer, upon illumination, the electric field is localized inside of the device, making capacitive coupling to the surrounding electrolyte impossible. It should be noted that the term “conductor” can often be used interchangeably with “metal,” however, it may be a highly-doped semiconductor (indium tin oxide for example), or a conducting polymer–therefore we use the general term “conductor” here.

In extended/exposed architectures, as shown in Figure 1C, the lateral separation of the metal from the organic layer dictates the localization of the current flow that is generated across the electrolytic solution. The semiconductor/electrolyte interface is the charge-generating electrode, while the exposed metal/electrolyte interface acts as the return electrode. As in the case of a traditional wired stimulation electrode, optoelectronic stimulation devices must consider both the stimulation and the return electrodes. The terms *return electrode* or *reference electrode* often appear in the neuromodulation literature, though these terms do not mean the same thing as in the electrochemical literature. Return or reference electrode refers to the electrode with respect to which a given potential or current at the primary stimulation electrode is applied, therefore for most purposes, this term identifies the electrical ground. In the case of self-contained optoelectronic devices, the concept of the return electrode must be carefully considered, as due to the limited size/geometry of such devices, the electrochemical current induced will be spatially confined by the two electrodes. Such optoelectronic devices with a two-electrode architecture can be either capacitive, where the photocarriers generated by the semiconductor component lead to the build-up to two oppositely-charged electrical double layers or photofaradaic where both electrodes of the device catalytically support faradaic reactions (Figure 1, bottom). The latter case resembles the “artificial leaf” concept developed by Reece et al. (2011) and Surendranath et al. (2012). For a sustained faradaic current to be present, both cathode and anode of the device must be suitably catalytic to support the given reactions, and the total photovoltage generated by the semiconductor component introduces the fundamental thermodynamic constraint on what faradaic reactions will be possible or not. Thus, it must be considered that a sustained photoelectrocatalytic cycle is not that likely due to limited voltage and non-ideal catalytic interfaces. The final aspect in determining faradaic vs. capacitive phenomena is dynamics—a sustained photocurrent at long time scales must be attributed to a faradaic reaction, but it may remain obscured by capacitive behavior for durations shorter than several capacitive time constants. In the neurostimulation field, the key figure of merit is the electrochemical charge density, defined as the integral of current density over a phase of a stimulus waveform (McCreery et al., 1990). Reports give threshold values of charge density for reproducible generation of action potentials (APs). For *in vivo* stimulation of APs on peripheral nerves, charge densities in the range of 2–50 μC/cm^2^ are used (McCreery et al., 1990; Cogan et al., 2017; Günter et al., 2019). Generally, the larger the stimulation electrode, the lower the necessary charge density for eliciting AP generation will be. For retinal stimulation, smaller area microelectrodes are desired to achieve a high spatial resolution of stimulation. For retinal ganglion cell stimulation with microelectrodes, action potential stimulation thresholds have been reported in the range of 0.05 mC/cm^2^ to roughly 1 mC/cm^2^, with thresholds declining for larger electrode sizes (Sekirnjak et al., 2006). While seemingly counterintuitive, this is due to current being injected over a larger area producing a transient voltage perturbation over a larger region of solution, thereby being able recruit more cells into a response.

## Quantifying Photocapacitive Vs. Photofaradaic Current

It is not immediately clear how to benchmark the performance of autonomous “Type C” devices designed for optoelectronic stimulation. Two- or three-electrode photoelectrochemical measurements of the conductor/semiconductor device stack can be useful to evaluate the electrolytic capacitance of the photoelectrode, photocurrent magnitude, and possibility of faradaic reactions. However, such measurements do not faithfully reproduce conditions where the device itself is wireless and electrically floating. Conventional electrochemical characterization techniques utilizing potentiostats are also not ideal considering that light pulses for neurostimulation are intended to last in the range 0.05–10 ms, showing relatively fast dynamics. Accordingly, we have as a rule adopted measuring this dynamic electrochemistry with an oscilloscope, measuring between the rear electrode and an electrolytic solution contacted with an Ag/AgCl electrode. Such a measurement can correctly resolve the charging/discharging dynamics. Nevertheless, this configuration still does not reflect the realistic “floating” conditions. Here we present a method to interrogate the photocurrents between the primary photoelectrode and rear conductor directly.

The device we test is an organic optoelectronic stimulator our group has recently introduced, called the organic electrolytic photocapacitor (OEPC). The OEPC was designed as a “Type C” device (see Figure 1C), employing a small-molecule evaporated semiconductor donor/acceptor (*a.k.a.* P/N) bilayer as the photocharge generation component. OEPC devices have been demonstrated to successfully generate action potentials in cultured neurons and in explanted embryonic chicken retinas, using red light (660 nm) pulses of 1–5 ms length. We have recently evaluated more optimized OEPC devices where it was possible to measure the photoinduced activation of K^+^ channels in single-cell voltage-clamp experiments (Jakešová et al., 2019). These experiments clearly show the strong depolarization in the range of 20–40 mV which OEPC devices can induce in cell membranes at a distance of several micrometers away from the semiconductor-electrolyte interface. Such large membrane voltage perturbations substantiate findings of action potential generation using the OEPC platform. In our previous published studies a photocapacitive mechanism was hypothesized on the basis of charge balance between cathodic and anodic charging pulses when measuring photocharging between the back electrode and a non-polarizable electrode in solution; the transient nature of these currents, which decay within a few ms of the pulse onset without displaying a sustained faradaic current, and the observation of reproducible and stable stimulation.

### Split-Electrode Photocurrent Response Measurement Method

To measure the photocurrent in a realistic “floating” OEPC device, we created a split bottom electrode architecture (Figure 2A). One conductor layer has the PN semiconductor layer on it, while the other is the blank back conductor. The “counter electrode” is 100 microns away from the “photoelectrode.” For these measurements, we fabricated a device consisting of a semitransparent gold back electrode topped by a PN charge-generating layer [30 nm metal free phthalocyanine (H_2_PC)/30 nm *N,N’*-dimethyl perylenetetracarboxylic diimide (PTCDI)]. An elastomer (PDMS, Sylgard 184) block with a well confines the electrolyte above the two electrodes, while with the help of microprobes, the two electrodes are short-circuited externally by a low-impedance high-bandwidth current amplifier (Figure 2B). The device was optically excited with a 630 nm LED driven light pulses. Temporally-resolved currents generated by the OEPC during the light pulse and delivered to the conductor back-electrode were measured to help resolve the nature of the processes taking place. If both the conductor/PN and the bare conductor electrodes can sustain respective cathodic/anodic faradaic reactions, a faradaic current should persist over long periods. In case the electrodes are fully capacitive and cannot sustain faradaic reactions, the current should exhibit a capacitive peak and decay once the electrical double layers fully charge. At the end of the light pulse, the double layers will discharge. The charge delivered during charging and discharging, i.e., time integrals under the current trace, for times significantly longer than the stimulation duration, should cancel out. If in fact both electrodes can support a low level of faradaic reactions, current traces will show a capacitive charging peak upon initiation of the light pulse followed by a sustained non-zero photocurrent. This current can only originate if faradaic processes are present on both of the split electrodes. Thus, the total current delivered by a photocapacitor can have both capacitive and a faradaic component. By integrating the current measured from the beginning of the light stimulation until a time significantly longer than the capacitive time constant *τ* after the end of the light pulse, we can test for charge conservation. The closer the charge is to zero, the more capacitive the device is. If there is any unaccounted charge left after the integration, the missing charge can be associated with faradaic reactions:

$$\int_{0}^{T\gg \tau }i\,\left(t\right)\,dt=\int_{0}^{T\gg \tau }i_{F}\,\left(t\right)+i_{C}\,\left(t\right)\,d\,t=\int_{0}^{T\gg \tau }i_{F}\,\left(t\right)\,dt+\int_{0}^{T\gg \tau }i_{C}\,\left(t\right)\,dt=Q_{F}+0$$

Overall current will be limited by the higher impedance electrode. Faradaic reactions will be controlled by the overpotential (i.e., kinetic barrier) of the given electrode, meaning that the capacitive vs. faradaic nature of the device can be tuned by the selection of the materials comprising the electrodes. Another important consideration is the open-circuit photovoltage of the PN structure. If the conductor chosen for a back electrode does not support electrochemical reactions within the open-circuit potential window of the OEPC, faradaic process on the charge-generating conductor/PN electrode may also not take place, since the redox processes on the two electrodes could not be balanced. On the other hand, if a perfectly non-polarizable electrode such as Ag/AgCl in Cl^–^ containing electrolyte is used for a back electrode, the faradaic reactions on the photocharge-generating electrode will not be hindered.

## Results and Discussion

The device was exposed to a series of light pulses of durations between 25 μs and 50 ms to study the charging dynamics (Figure 2C). A 1 s off time was held between consecutive pulses to make sure that the devices have enough time to reestablish the thermodynamic equilibrium. For each pulse duration, a current trace was measured and integrated during the light pulse duration and the following discharge time. Before each measurement, a dark current was recorded for the same duration, averaged, and the current zero offset was adjusted. For each pulse duration, a total integrated charge *Q*, as well as the charge delivered over positive (*Q*_+_) and the negative (*Q*_–_) phase of the current were evaluated. The rate of change of the total, un-accounted charge left after the integration of the current traces of different durations, d*Q*/d*t* (Figure 2D), was plotted as well. This value can tentatively be attributed to the faradaic component of the total current delivered by the photocapacitor.

In the case of pulses lasting longer than 10 ms (Figure 2E) capacitive charge and discharge pulses are clearly evident, with the capacitive time constant τ = *RC* of about 1 millisecond. Even though the total integrated charge *Q* does not completely cancel out, there is no sustained faradaic current *dQ*/*dt* for longer time durations. However, in the case of pulses significantly shorter than the time constant τ, a negative current discharge pulse was not observed at all (Figure 2C). A progressively more-pronounced discharge pulse was observed for pulse durations between 200 μs and 1 ms. This may lead one to believe that the faradaic process is present for short pulses, while for longer pulses the process is more capacitive in nature. While this may be the case, it appears that this is a measurement artifact. Voltage pulses significantly shorter than the time constant τ = *RC* of a *RC* circuit and thus possessing a significant high frequency content can pass through the capacitor acting as a high-pass filter virtually unchanged, without eliciting a pronounced capacitive discharge peak. During the short duration of the pulse of the order of 0.1 *RC*, the photocapacitor doesn’t have the time to charge up significantly, even though the charging current can be of significant magnitude. This small amount of charge discharges after the end of the light stimulation, but following the dynamics of the *RC* circuit—thus it takes 5*RC* for > 99% of the charge to be discharged, which can be > 50 times the stimulation pulse duration, meaning that the discharge current can be extremely small, and present over a long time. This presents an experimental difficulty, since it is necessary to measure the current trace both with high temporal and current resolution, and to eliminate the measurement offset completely. Even though we used a high bandwidth current amplifier, and high dynamic range and high-speed oscilloscope, with paying attention to zero the offset before every measurement, it is likely that a significant portion of the discharge current was lost in the measurement noise, which thus yielded a false faradaic current as a result. For verification, a “dry” photodiode device was manufactured, with the same device architecture as a H_2_PC/PTCDI photocapacitor, but with added top titanium metal contact. A 47 nF ceramic capacitor was connected in series with this photodiode and the current traces were measured with light pulses of different length. Qualitatively the same behavior as in a “wet” photocapacitor was observed, with sub-0.1RC pulses showing no or negligible discharge current. Therefore this is an important artifact that should be taken into consideration when measuring dynamic electrochemical currents.

Another contribution to the short-time scale non-balanced current effect may be the “dark charging” of the double layers between the light pulses. In darkness after the thermodynamic equilibrium is established, the gold/PN electrode was observed to have a positive potential vs. the bare gold electrode (measured using an electrometer), while the opposite polarity was observed in short-circuit conditions during the excitation by a light pulse. This leads to the possible conclusion that in the dark the double layers of the device are charged slowly while establishing the thermodynamic equilibrium, and the device enters the light-pulse cycle pre-charged. The charge generation during the light pulse lowers the impedance of the PN junction by 3–4 orders of magnitude (Rand et al., 2018), and the charges at the opposite device electrodes can discharge, leading to the observed non-balanced current pulse at short times and the reversal of the polarities of the electrodes. The equilibrium during which the electrical double layers get charged is established over relatively long times, on the order of seconds. These low “dark” charging currents, however, are below the amplifier’s noise limit, so they cannot be reliably measured over such long-time spans.

This shows that the measured charge dynamics of the floating photocapacitor corresponds to the case of a *RC* circuit, charged by a photovoltaic *PN* element. In our model, the process is capacitive in nature, even for the short pulses, and shows that the device can get a boost in delivered current from its pre-charged state in case of short light pulses separated by much longer durations of darkness, as is typically the case in electrical neuro-stimulation.

From the split-electrode measurement, it is possible to resolve the dynamics of the photocurrents and conclusively establish that the OEPC devices do not source stable faradaic current. An unexpected phenomenon observed at the shortest measured time scales of tens of microseconds, may be explained by the photogeneration process first discharging two preexisting double layers of the opposite polarity, which are the result of the thermodynamic equilibrium of the device/electrolyte structure in the dark.

## Future Outlook for Organic Optoelectronic Stimulation Devices

Organic semiconductors hold the promise to enable ultrathin and biocompatible bio-interfacing devices for cellular photostimulation. At present this field is still in its infancy. Success depends on several critical factors. The first is photocurrent optimization and the resulting charge density. If the goal is to mimic and replace conventional electrical stimulation protocols, organic semiconductor devices must be engineered to deliver charge densities in the range of > 1 μC/cm^2^ over timescales of 100–5,000 μs. The second is operational robustness and long-term safety. Promising indications for biocompatibility and stability for organic devices exist, however long-term stability and efficacy must be established. Finally, photochemical and photofaradaic reactions remain to be fully understood. The recent reports on reactive oxygen species generation by organic semiconductors represent on one hand a caveat for deployment of such stimulation devices, on the other hand there are also many opportunities for on-demand ROS delivery. Therefore, moving forward demands careful characterization of the photochemical, photofaradaic, and photocapacitive properties of organic materials and devices. In this perspective, we attempted to bring these concepts to the forefront and to offer a measurement strategy to resolve the photofaradaic vs. photocapacitive issue in realistic conditions.

## Data Availability Statement

All datasets generated for this study are included in the article/supplementary material.

## Author Contributions

EG and VÐ conceived the research idea. EG and YH supervised and coordinated the research. VÐ fabricated and tested all the devices, with fabrication and measurement assistance from LM and DR. EG wrote the first draft of the manuscript. VÐ, EG, DR, and YH wrote sections of the manuscript. All authors contributed to manuscript revision, read and approved the submitted version.

## Conflict of Interest

The authors declare that the research was conducted in the absence of any commercial or financial relationships that could be construed as a potential conflict of interest.

## References

[B1] Albert E. S. Bec J. M. Desmadryl G. Chekroud K. Travo C. Gaboyard S. (2012). TRPV4 channels mediate the infrared laser-evoked response in sensory neurons. *J. Neurophysiol.* 107 3227–3234. 10.1152/jn.00424.2011 22442563

[B2] Antognazza M. R. Aziz I. A. Lodola F. (2019). Use of exogenous and endogenous photo-mediators as efficient ROS modulation tools: results & perspectives for therapeutic purposes. *Oxid. Med. Cell. Longev.* 2019 1–24. 10.1155/2019/2867516 31049131 PMC6462332

[B3] Bellani S. Antognazza M. R. Bonaccorso F. (2018). Carbon-based photocathode materials for solar hydrogen production. *Adv. Mater.* 31:1801446. 10.1002/adma.201801446 30221413

[B4] Bellani S. Ghadirzadeh A. Meda L. Savoini A. Tacca A. Marra G. (2015). Hybrid organic/inorganic nanostructures for highly sensitive photoelectrochemical detection of dissolved oxygen in aqueous media. *Adv. Funct. Mater.* 25 4531–4538. 10.1002/adfm.201500701

[B5] Carvalho-de-Souza J. L. Treger J. S. Dang B. Kent S. B. H. Pepperberg D. R. Bezanilla F. (2015). Photosensitivity of neurons enabled by cell-targeted gold nanoparticles. *Neuron* 86 207–217. 10.1016/j.neuron.2015.02.033 25772189 PMC4393361

[B6] Chowdhury P. Fortin P. Suppes G. Holdcroft S. (2016). Aqueous photoelectrochemical reduction of anthraquinone disulfonate at organic polymer films. *Macromol. Chem. Phys.* 217 1119–1127. 10.1002/macp.201500440

[B7] Cogan S. F. Ludwig K. A. Welle C. G. Takmakov P. Clinic M. Spring S. (2017). Tissue damage thresholds during therapeutic electrical stimulation. *J. Neural Eng.* 13:021001. 10.1088/1741-2560/13/2/021001 26792176 PMC5386002

[B8] Gamper N. Zaika O. Li Y. Martin P. Hernandez C. C. Perez M. R. (2006). Oxidative modification of M-type K + channels as a mechanism of cytoprotective neuronal silencing. *EMBO J.* 25 4996–5004. 10.1038/sj.emboj.7601374 17024175 PMC1618113

[B9] Gryszel M. Markov A. Vagin M. Głowacki E. D. (2018a). Organic heterojunction photocathodes for optimized photoelectrochemical hydrogen peroxide production. *J. Mater. Chem. A* 6 24709–24716. 10.1039/C8TA08151D

[B10] Gryszel M. Rybakiewicz R. Głowacki E. D. (2019). Water-soluble organic dyes as molecular photocatalysts for H2O2 evolution. *Adv. Sustain. Syst.* 3:1900027 10.1002/adsu.201900027

[B11] Gryszel M. Sytnyk M. Jakesova M. Romanazzi G. Gabrielsson R. Heiss W. (2018b). General observation of photocatalytic oxygen reduction to hydrogen peroxide by organic semiconductor thin films and colloidal crystals. *ACS Appl. Mater. Interfaces* 10 13253–13257. 10.1021/acsami.8b01295 29624365

[B12] Günter C. Delbeke J. Ortiz-Catalan M. (2019). Safety of long-term electrical peripheral nerve stimulation: review of the state of the art. *J. Neuroeng. Rehabil.* 16:13. 10.1186/s12984-018-0474-8 30658656 PMC6339286

[B13] Huang Y. Y. Nagata K. Tedford C. E. Mccarthy T. Hamblin M. R. (2013). Low-level laser therapy (LLLT) reduces oxidative stress in primary cortical neurons in vitro. *J. Biophotonics* 6 829–838. 10.1002/jbio.201200157 23281261 PMC3651776

[B14] Jakešová M. Apaydin D. H. Sytnyk M. Oppelt K. Heiss W. Sariciftci N. S. (2016). Hydrogen-bonded organic semiconductors as stable photoelectrocatalysts for efficient hydrogen peroxide photosynthesis. *Adv. Funct. Mater.* 26 5248–5254. 10.1002/adfm.201601946

[B15] Jakešová M. Ejneby M. S. Ðerek V. Schmidt T. Gryszel M. Brask J. (2019). Optoelectronic control of single cells using organic photocapacitors. *Sci. Adv.* 5:eaav5265. 10.1126/sciadv.aav5265 30972364 PMC6450690

[B16] Jiang Y. Carvalho-de-Souza J. L. Wong R. C. S. Luo Z. Isheim D. Zuo X. (2016). Heterogeneous silicon mesostructures for lipid-supported bioelectric interfaces. *Nat. Mater.* 15 1023–1030. 10.1038/nmat4673 27348576 PMC5388139

[B17] Jiang Y. Parameswaran R. Li X. Carvalho-de-Souza J. L. Gao X. Meng L. (2019). Nongenetic optical neuromodulation with silicon-based materials. *Nat. Protoc.* 14 1339–1376. 10.1038/s41596-019-0135-9 30980031 PMC6557640

[B18] Lim J. B. Langford T. F. Huang B. K. Deen W. M. Sikes H. D. (2016). A reaction-diffusion model of cytosolic hydrogen peroxide. *Free Radic. Biol. Med.* 90 85–90. 10.1016/j.freeradbiomed.2015.11.005 26561774

[B19] Lin Y. Li Y. Zhan X. (2012). Small molecule semiconductors for high-efficiency organic photovoltaics. *Chem. Soc. Rev.* 41 4245–4272. 10.1039/c2cs15313k 22453295

[B20] Lodola F. Martino N. Tullii G. Lanzani G. Antognazza M. R. (2017). Conjugated polymers mediate effective activation of the Mammalian ion channel transient receptor potential vanilloid 1. *Sci. Rep.* 7:8477. 10.1038/s41598-017-08541-6 28814817 PMC5559550

[B21] Lodola F. Rosti V. Tullii G. Desii A. Tapella L. Catarsi P. (2019). Conjugated polymers optically regulate the fate of endothelial colony-forming cells. *Sci. Adv.* 5:eaav4620. 10.1126/sciadv.aav4620 31598549 PMC6764832

[B22] McCreery D. B. Agnew W. F. Yuen T. G. H. Bullara L. (1990). Charge density and charge per phase as cofactors in neural injury induced by electrical stimulation. *IEEE Trans. Biomed. Eng.* 37 996–1001. 10.1109/10.102812 2249872

[B23] Migliaccio L. Gryszel M. Ðerek V. Pezzella A. Głowacki E. D. (2018). Aqueous photo(electro)catalysis with eumelanin thin films. *Mater. Horizons* 5 984–990. 10.1039/C8MH00715B

[B24] Mitraka E. Gryszel M. Vagin M. Jafari M. J. Singh A. Warczak M. (2019). Electrocatalytic production of hydrogen peroxide with Poly(3,4-ethylenedioxythiophene) electrodes. *Adv. Sustain. Syst.* 3:1800110 10.1002/adsu.201800110

[B25] Moros M. Lewinska A. Onorato G. Antognazza M. R. Di Maria F. Blasio M. (2018). Light-triggered modulation of cell antioxidant defense by polymer semiconducting nanoparticles in a model organism. *MRS Commun.* 155 1–8. 10.1557/mrc.2018.104

[B26] Rand D. Jakešová M. Lubin G. Vebraite I. David-Pur M. Ðerek V. (2018). Direct electrical neurostimulation with organic pigment photocapacitors. *Adv. Mater.* 30:1707292. 10.1002/adma.201707292 29717514

[B27] Reece S. Y. Hamel J. A. Sung K. Jarvi T. D. Esswein A. J. Pijpers J. J. H. (2011). Wireless solar water splitting using silicon-based semiconductors and earth-abundant catalysts. *Science* 334 645–648. 10.1126/science.1209816 21960528

[B28] Sekirnjak C. Hottowy P. Sher A. Dabrowski W. Litke A. M. Chichilnisky E. J. (2006). Electrical stimulation of mammalian retinal ganglion cells with multielectrode arrays. *J. Neurophysiol.* 95 3311–3327. 10.1152/jn.01168.2005 16436479

[B29] Shapiro M. G. Homma K. Villarreal S. Richter C.-P. Bezanilla F. (2012). Infrared light excites cells by changing their electrical capacitance. *Nat. Commun.* 3:736. 10.1038/ncomms1742 22415827 PMC3316879

[B30] Suppes G. Ballard E. Holdcroft S. (2013). Aqueous photocathode activity of regioregular poly(3-hexylthiophene). *Polym. Chem.* 4 5345–5350. 10.1039/c3py00143a

[B31] Surendranath Y. Bediako D. K. Nocera D. G. (2012). Interplay of oxygen-evolution kinetics and photovoltaic power curves on the construction of artificial leaves. *Proc. Natl. Acad. Sci. U.S.A.* 109 15617–15621. 10.1073/pnas.1118341109 22689962 PMC3465448

[B32] Sytnyk M. Jakešová M. Litviòuková M. Mashkov O. Kriegner D. Stangl J. (2017). Cellular interfaces with hydrogen-bonded organic semiconductor hierarchical nanocrystals. *Nat. Commun.* 8:91. 10.1038/s41467-017-00135-0 28733618 PMC5522432

[B33] Tortiglione C. Antognazza M. R. Tino A. Bossio C. Marchesano V. Bauduin A. (2017). Semiconducting polymers are light nanotransducers in eyeless animals. *Sci. Adv.* 3:e1601699. 10.1126/sciadv.1601699 28138549 PMC5266477

[B34] Warczak M. Gryszel M. Jakešová M. Ðerek V. Głowacki E. D. (2018). Organic semiconductor perylenetetracarboxylic diimide (PTCDI) electrodes for electrocatalytic reduction of oxygen to hydrogen peroxide. *Chem. Commun.* 54 1960–1963. 10.1039/C7CC08471D 29323369

[B35] Węcławski M. K. Jakešová M. Charyton M. Demitri N. Koszarna B. Oppelt K. (2017). Biscoumarin-containing acenes as stable organic semiconductors for photocatalytic oxygen reduction to hydrogen peroxide. *J. Mater. Chem. A* 5 20780–20788. 10.1039/C7TA05882A PMC11976386 40213317

[B36] Willner I. Katz E. (2005). *Bioelectronics.* Weinheim: Wiley-VCH.

[B37] Zimmerman J. F. Tian B. (2018). Nongenetic optical methods for measuring and modulating neuronal response. *ACS Nano* 12 4086–4095. 10.1021/acsnano.8b02758 29727159 PMC6161493

